# Rapid Restriction Enzyme-Free Cloning of PCR Products: A High-Throughput Method Applicable for Library Construction

**DOI:** 10.1371/journal.pone.0111538

**Published:** 2014-10-31

**Authors:** Vijay K. Chaudhary, Nimisha Shrivastava, Vaishali Verma, Shilpi Das, Charanpreet Kaur, Payal Grover, Amita Gupta

**Affiliations:** 1 Department of Biochemistry, University of Delhi South Campus, Benito Juarez Road, New Delhi 110021, India; 2 Department of Microbiology, University of Delhi South Campus, Benito Juarez Road, New Delhi 110021, India; Federal University of Pelotas, Brazil

## Abstract

Herein, we describe a novel cloning strategy for PCR-amplified DNA which employs the type IIs restriction endonuclease BsaI to create a linearized vector with four base-long 5′-overhangs, and T4 DNA polymerase treatment of the insert in presence of a single dNTP to create vector-compatible four base-long overhangs. Notably, the insert preparation does not require any restriction enzyme treatment. The BsaI sites in the vector are oriented in such a manner that upon digestion with BsaI, a stuffer sequence along with both BsaI recognition sequences is removed. The sequence of the four base-long overhangs produced by BsaI cleavage were designed to be non-palindromic, non-compatible to each other. Therefore, only ligation of an insert carrying compatible ends allows directional cloning of the insert to the vector to generate a recombinant without recreating the BsaI sites. We also developed rapid protocols for insert preparation and cloning, by which the entire process from PCR to transformation can be completed in 6–8 h and DNA fragments ranging in size from 200 to 2200 bp can be cloned with equal efficiencies. One protocol uses a single tube for insert preparation if amplification is performed using polymerases with low 3′-exonuclease activity. The other protocol is compatible with any thermostable polymerase, including those with high 3′-exonuclease activity, and does not significantly increase the time required for cloning. The suitability of this method for high-throughput cloning was demonstrated by cloning batches of 24 PCR products with nearly 100% efficiency. The cloning strategy is also suitable for high efficiency cloning and was used to construct large libraries comprising more than 10^8^ clones/µg vector. Additionally, based on this strategy, a variety of vectors were constructed for the expression of proteins in *E. coli,* enabling large number of different clones to be rapidly generated.

## Introduction

The availability of genome sequences from a large number of organisms has created a wide-spread need to clone complete sets of open reading frames (ORFs) followed by the expression and purification of the encoded gene products in a high-throughput manner to examine the functions of genes and proteins in organisms. Using a variety of thermostable polymerases, the target ORFs can be efficiently amplified by polymerase chain reaction (PCR) with virtually no errors. However, high efficiency and high-throughput cloning of amplified products into a suitable expression vector still remains an arduous task.

An ideal high-throughput cloning method should include a minimal number of steps without the need for intermediate purification and preferably be performed in the same tube in which the gene-of interest was amplified. For such cloning, (i) the reagents used to prepare the vector and insert should not be proprietary and should be available from multiple sources; (ii) the vector should not self-ligate and the insert should not form concatemers, rather ligate to the vector in the desired orientation; (iii) the primers for amplification should carry only small additional sequences without the need for any modifications, and thereby only add a sequence encoding a few amino acids at the vector/insert junctions to serve as spacers, and finally; (iv) the vector design should be amenable to modifications such as incorporation of different tags to enable purification and/or enhance solubility, or changing the promoter, antibiotic selection marker and even the origin of replication.

Traditional methods such as restriction enzyme-based strategies cannot be considered suitable for the high-throughput cloning of a large set of different ORFs due to the presence of numerous restriction enzyme sites within the ORF sequences [1]. As an alternative, the commonly used TA cloning methodology is simple, but lacks directionality in cloning and requires the inserts to be amplified using enzymes that have template-independent terminal transferase activity; these polymerases have a low fidelity resulting in mutations in the amplified DNA [2]. Different formats of the ligation-independent cloning (LIC) method have been described for efficient high-throughput cloning, but rely on the annealing of complementary single-stranded DNA and capability of bacterial cells to repair the resulting gaps within double-stranded DNA. LIC involves amplification of genes of interest with primers containing an additional sequence of 15–20 bases, which is then used to create long cohesive ends on the amplified inserts by T4 DNA polymerase or exonuclease III treatment under controlled conditions *in vitro*. The long overhangs of these inserts are then annealed to the linearized vector that carries compatible long cohesive ends created by specific treatments, then without prior ligation, the vector-insert mixture is transformed into a bacterial host in which the nicks/gaps are filled [3]. The disadvantage of this method is the addition of unwanted amino acids at both ends of the expressed protein. Methods such as sequence and ligation-independent cloning (SLIC) have been described as an alternative to LIC; however, their cloning efficiency in the absence of a recombinase, RecA, is low [4]. Recently described methods like Seamless Ligation Cloning Extract, SLiCE, also require the addition of up to 42–52 bp to the insert to provide end homology in order to achieve a very high efficiency. In fact, inserts without end homology or less than 10 bp end homology did not yield any recombinant clones [5]. Recombination-based cloning methods including Gateway [6], [7], Creator [8] etc., have been developed for high-throughput cloning; however, all of these methods make use of long site-specific sequences for recombination that must be incorporated on either side of the ORF to enable its insertion into the desired vector. Moreover, these methods are expensive and impose restrictions in terms of the sequences and hosts. Commercially available cloning systems such as TOPO cloning [9], Infusion [10] etc., require specialized vectors and/or proprietary reagents to create cohesive ends in the vector and inserts. Recently, simple high-throughput cloning strategies based on type IIs restriction enzymes have been described [11], [12], but require the inserts to be amplified with primers carrying the restriction site of choice. However, the limitation of all type IIs restriction enzyme-based cloning is the possibility that the insert may contain the same internal restriction enzyme site, requiring the use of alternative strategies such as site-directed mutagenesis of the endogenous restriction site. Another major drawback of most of the strategies described above is the requirement to purify the PCR products prior to ligation to the vector.

In this paper, we present a highly versatile and efficient restriction enzyme-free cloning strategy for rapid and high-throughput cloning of PCR-amplified DNA fragments into the desired vector. We demonstrate the strategy is equally effective for cloning inserts of various sizes (0.5 kbp to 2.2 kbp) in single tube format suitable for high-throughput applications, and also for high efficiency cloning during the construction of genome-scale libraries (more than 10^8^ clones). Based on this strategy, several expression vectors were constructed and employed for cloning hundreds of mycobacterial genes to produce proteins containing different tags. The versatility and high efficiency of this strategy has been extended by constructing vectors for a number of different applications including phage display of gene-fragments and construction of mouse antibody libraries.

## Materials and Methods

### Construction of vectors

**Figure 1:**
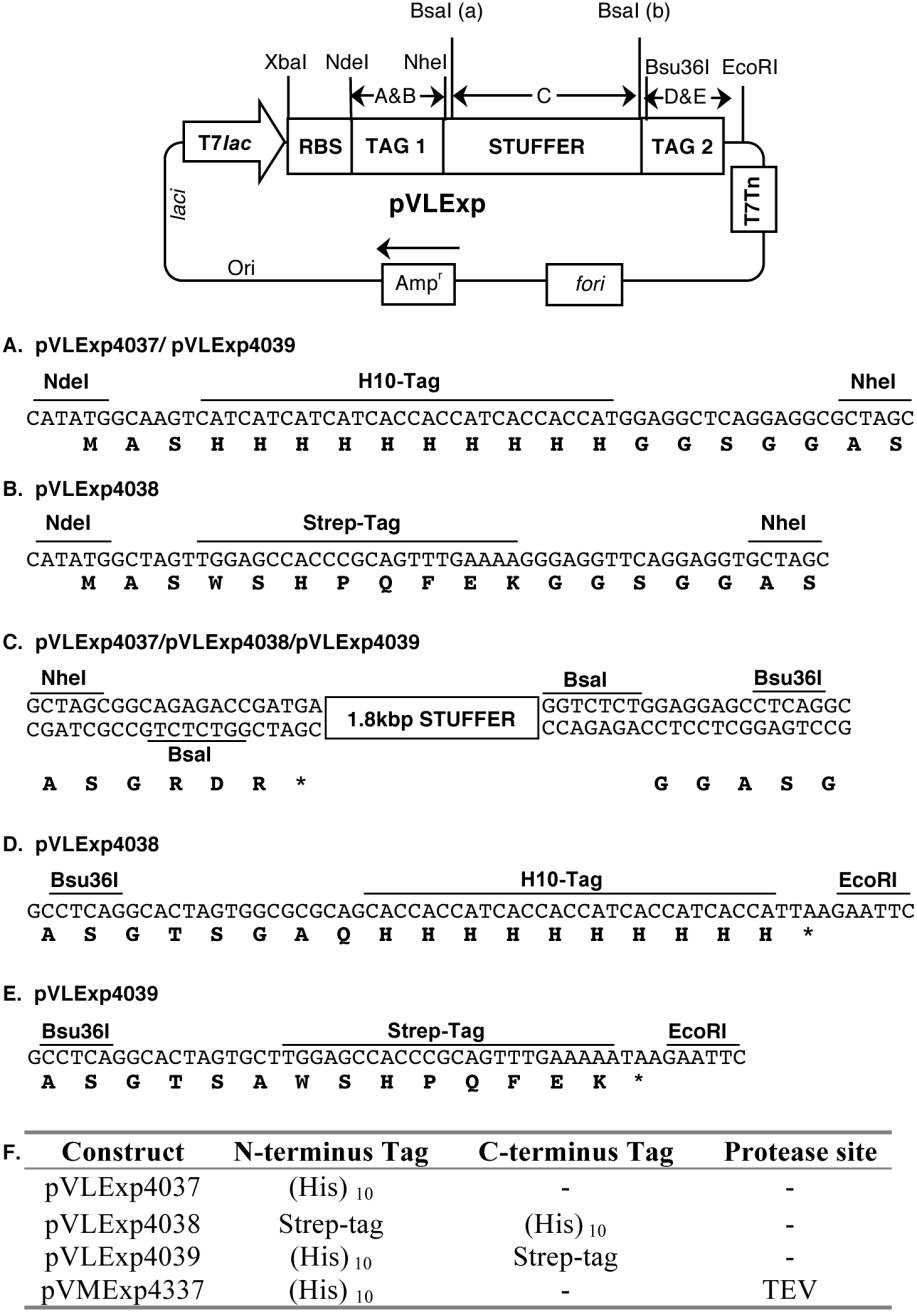
Diagrammatic representation of pVLExp4037/4038/4039 vectors. Only relevant genes and restriction sites are shown. The maps are not to scale. T7*lac*, T7 promoter lac operator; RBS ribosome-binding site; T7Tn, T7 transcription terminator; *fori*, origin of replication of filamentous phage; Amp^r^, β lactamase gene; Ori, Col E1 origin of replication; *laci*, lac repressor encoding gene rop/rom; Stuffer, 1.8 kbp stuffer flanked by BsaI sites. The encoded amino acids are shown in single letter code (bold) below the nucleotide sequence (A–E). F, shows the summary of vectors containing different fusion tags and protease sites.

The expression vector pVNLEBAP1306 [13] containing a backbone obtained from pET11a [14] was modified by oligonucleotide-directed mutagenesis to delete a 1.32 kbp fragment between the *laci* and *rop* genes to derive the pVLExp vector backbone (Fig. 1 & Fig. S7). A stuffer flanked by two BsaI sites in the appropriate orientations for cloning and containing other unique restriction sites, and the sequences encoding fusion tags was introduced between the NdeI and EcoRI sites. The NdeI site harboring the initiation codon (CAT**ATG**) was followed by a sequence encoding an N-terminal tag (Tag1), NheI site, 5′ inverted BsaI site, 1.8 kbp stuffer, 3′ BsaI site followed by a Bsu36I restriction site, the sequence encoding a C-terminal tag (Tag2) and the stop codon (TAA). Digestion of the vector with BsaI linearizes the vector, creating a 5′-GCCG-3′ overhang close to the 3′ end of the promoter and 5′-GGAG-3′ overhang close to the EcoRI site, and removes the stuffer so that the BsaI recognition sites are lost upon ligation with the insert.

The vector for cloning was prepared by digesting 10 µg plasmid DNA in a total volume of 400 µl with 400 units of BsaI (New England Biolabs, Ipswich, MA, USA) added in four aliquots during the 4 h incubation at 50°C. The digested DNA was extracted with phenol: chloroform, ethanol precipitated, resuspended in 0.1X TE and then separated on 1.2% Sea Plaque GTG agarose (Lonza, Rockland, ME, USA) to purify the linearized vector from the stuffer fragment using the Qiaquick Gel Extraction kit (Qiagen, Hilden, Germany).

### Insert preparation

**Figure 2:**
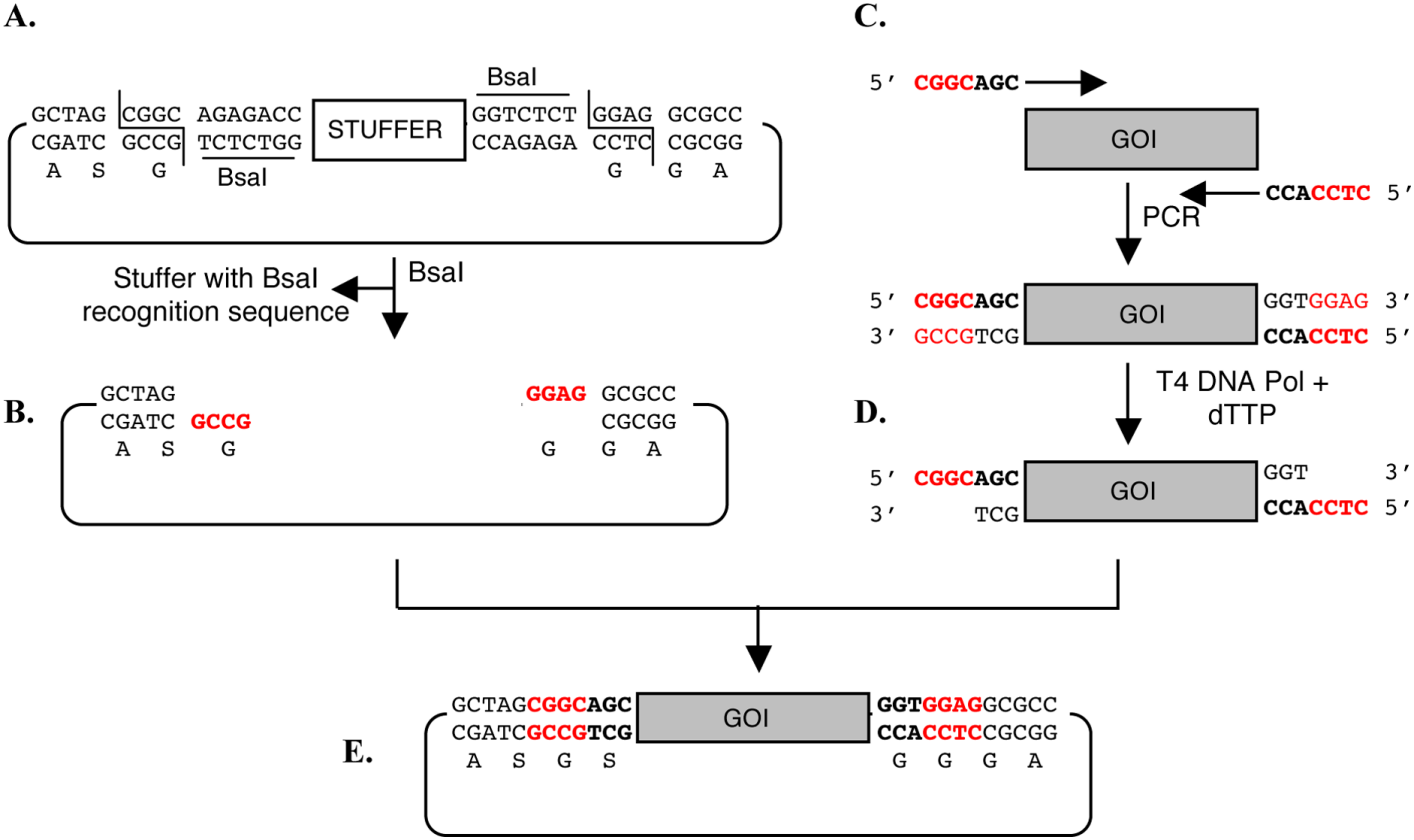
Cloning strategy. The vector contains two appropriately oriented BsaI sites (A) upon digestion with BsaI linearized vector is obtained with ends having 4-base 5′-overhangs (B) shown in red. The recognition sequence of restriction enzyme BsaI are underlined and the cleavage site is marked. The Gene Of Interest (GOI) is amplified using two gene-specific primers with 7-base long additional sequence at the 5′ end (C) shown in bold. Treatment of PCR product with T4 DNA polymerase and dTTP produces two different four-base overhangs that are complementary to two ends of the linearized vector shown in red (D). The ligation results in direction cloning of the insert into the vector (E).

The forward and reverse primers used for amplification of genes of interest carried 5′-CGGCAGC and 5′-CTCCACC, respectively, as seven base-long extensions in addition to the approximately 20–26 base-long gene-specific sequence (Fig. 2C). The sequences of the primers used to amplify the different mycobacterial genes for the initial optimizations are shown in Table S1. Primers used for amplification were either molecular biology grade (without any purification) or HPLC-purified (IBA GmbH, Goettingen, Germany).

For initial optimizations, PCR was performed in 100 µl reactions containing 10 ng of plasmid carrying the test genes or 100 ng of *Mycobacterium tuberculosis (Mtb)* genomic DNA as a template, as well as 200 µM dNTPs, 50 pmoles each of the forward and reverse primers and 2 U of Expand High Fidelity PCR System (Expand HF polymerase; Roche, Mannheim, Germany). The amplification steps involved initial denaturation at 95°C for 3 min followed by 25 cycles of denaturation at 95°C for 30 sec, annealing at 55°C for 30 sec, polymerization at 72°C for variable periods of time depending upon the size of the genes (as per the manufacturer’s instructions) and 2 sec extension in each cycle followed by a final polymerization at 72°C for 4 min. Five microlitres of each reaction was analyzed by agarose gel electrophoresis to estimate the amount of PCR product; the remaining product was processed by the following methods to prepare the inserts for cloning.

### Single tube method

Ten microlitres of PCR product was mixed with 1 µl Exonuclease I-Shrimp Alkaline Phosphatase (Exo-SAP; Affymetrix, Santa Clara, CA, USA) in a 0.2 ml PCR tube, vortexed, centrifuged and incubated in a thermocycler programmed for 60 min incubation at 37°C followed by heat inactivation at 80°C for 20 min, then the tube was held at 4°C. One microlitre of dTTP (20 mM; 1.7 mM final concentration) was added to each tube containing the Exo-SAP treated inserts, mixed well by mild vortexing followed by brief centrifugation at 4°C, and 1.5 units (0.5 µl) of T4 DNA polymerase (New England Biolabs) were added. The contents were mixed well, centrifuged at 4°C and then placed in a thermocycler for incubation at 15°C for 60 min followed by heat inactivation at 75°C for 20 min.

Ligation was performed in a total volume of 10 µl containing 1 µl of 10X ligation buffer (New England Biolabs), 25 ng BsaI-HF-digested vector, 1 µl of T4 DNA Polymerase-treated insert (2–5 fold molar excess relative to the vector) and 1.0 unit of T4 DNA ligase (Roche) for 16 h at 16°C followed by 1 h at 37°C and heat inactivation at 65°C for 10 min. For electroporation, the ligation mixture was diluted ten-fold in water and then 1 µl of the diluted ligation sample (containing 250 pg vector equivalent) was electroporated into 25 µl of *E. coli* competent cells [BL21 (DE3) RIL, Agilent Technologies, CA, USA; efficiency 3–5×10^8^/µg]. The resulting recombinants were analyzed by colony PCR followed by sequencing using an ABI 3730 DNA sequencer (Life Technologies Corporation, USA). To check for protein expression, individual colonies were inoculated into 150 µl MDAGAmp_100_ medium [15] in a 96 well microtiter plate and cultured at 37°C with shaking for 3 h, and an aliquot of each suspension was subjected to PCR. Additionally, 20 µl of the cultures were transferred to 96-well deep well plate (2 ml capacity) containing 0.6 ml ZYM5052Amp_100_ auto-inducing medium [15] and incubated at 30°C with shaking at 300 rpm. After 16 h of induction, 50 µl of each culture was mixed with 50 µl of 2X Laemmli reducing dye and analyzed by SDS-PAGE using gradient gel (8–20%) electrophoresis and the bands were visualized using Coomassie Brilliant blue R-250 staining.

### Rapid single tube strategy

To further optimize the single tube strategy for rapid cloning with reduced incubation times for T4 DNA polymerase treatment and ligation, three mycobacterial genes of various sizes [*Rv1886c* (Ag85B), *Rv1908c* (MPT64) and *Rv3763* (19 KDa)] were amplified using the gene-specific primers containing specific 7-base extensions (Table S1) in a total volume of 50 µl. The inserts were processed as described above for the single tube strategy, except that the time of incubation of the inserts with T4 DNA polymerase to generate 5′ overhangs in the presence of dTTP was systematically reduced from 60 min to 5 min at 15°C followed by heat inactivation at 75°C for 20 min. The timing of the ligation was optimized by testing 60, 30 or 15 min incubation at room temperature (25°C) followed by heat inactivation at 65°C for 10 min. Then, 1 µl of the ligation mixtures were directly electroporated into 25 µl of BL21 (DE3) RIL electrocompetent cells (efficiency 2×10^8^/µg supercoiled plasmid DNA) and plated onto non-inducing MDAGAmp_100_plates. Furthermore, five different scFv genes cloned into phage display vectors (carrying ampicillin antibiotic resistance genes) were PCR amplified and sub-cloned into an Arabinose promoter-based expression vector pVMAAscFvclo 0001(carrying a ampicillin antibiotic resistance gene) using the rapid single tube method, and the resulting recombinants were analyzed by colony PCR and sequencing as described above.

### Column method

For PCR products obtained using high fidelity polymerases [16] such as PfuUltra II Fusion HS DNA Polymerase, Herculase II Fusion DNA Polymerase etc. (error rate ∼4.3×10^−7^) that have very high 3′ to 5′ exonuclease activity, the PCR-amplified product needs to be purified using a column to remove the polymerase, excess dNTPs and primers.

Therefore, the remaining ∼85 µl of the amplified products (out of initial 100 µl) were column purified using the Qiaquick PCR purification kit (Qiagen) as per the manufacturer’s instructions and the DNA was eluted in 60 µl of 1X TE. For treatment with T4 DNA polymerase, 10 µl of purified PCR product was mixed with 1 µl of 10X NEB 2 buffer (New England Biolabs), 0.1 µl of bovine serum albumin (BSA) and 1.0 µl of 20 mM dTTP (1.7 mM final concentration), briefly centrifuged at 4°C and 0.5 µl (1.5 units) of T4 DNA polymerase was added to obtain a total volume of 12.6 µl. The tubes were incubated at 15°C for 60 min, followed by heat inactivation of the enzyme at 75°C for 20 min, ligation and electroporation were performed as described above for the single tube strategy, and the resulting clones were analyzed by colony PCR and sequencing.

### Preparation of inserts for library-scale cloning

The PCR products (preparation described in Fig. S8) were purified using the Qiaquick PCR purification kit following the manufacturer’s instructions and subjected to agarose gel electrophoresis to estimate the DNA concentration. In a 1.5 ml microfuge tube, 5 µg of the purified PCR product was mixed with 10 µl of 10X NEB 2 buffer, 2.5 µl of 20 mM dTTP (500 µM dTTP final concentration) and the volume was adjusted to 98.5 µl with H_2_O. While keeping the tube on ice, 1.5 µl of T4 DNA polymerase (3 U/µl) was added, the contents were mixed by mild vortexing, centrifuged at 4°C, incubated at 15°C for 60 min and the enzyme was inactivated by adding 5 volume of Qiaquick buffer PB. The inserts were further column purified and analyzed by agarose gel electrophoresis. Then, 10 µl ligation reactions were set-up in multiple tubes, each containing 1 µl of 10X ligation buffer, 100 ng BsaI-digested vector, 25–100 ng T4 DNA polymerase-treated purified inserts (2–8 fold molar excess) and 1.0 unit of T4 DNA ligase (Roche) and incubated for 16 h at 16°C, 60 min at 37°C and heat inactivated at 65°C for 10 min. For electroporation, the contents of ligation reaction were pooled and 4 µl ligation mixture was directly electroporated into 100 µl of competent *E. coli* cells (efficiency 7–8×10^9^/µg) and several such electroporations were performed to obtain a library of ∼10^8^ clones.

## Results

### Concept of the cloning strategy

The cloning strategy described here is based on a combination of techniques involving the use of the type IIs restriction endonuclease BsaI to create a linearized vector with 4 base-long 5′-overhangs, and T4 DNA polymerase treatment of the insert in the presence of a single dNTP to create vector-compatible 4 base-long overhangs. The cohesive ends produced in the vector and the insert are non-compatible and non-palindromic in *cis* to prevent vector self-ligation or insert concatemerization, but allow directional ligation of the insert to the vector (Fig. 2).

The BsaI sites in the vector are oriented in such a manner that the stuffer, along with the recognition sequences of both sites, is removed upon digestion with BsaI. Thus, digestion of the vector with BsaI produces a linearized vector with two four base-long overhangs: 5′-GCCG-3′ on one end close to the initiation codon and 5′-GGAG-3′ at the other end near to the EcoRI site (Fig. 2A & 2B). The sequence of the four base-long overhangs produced by BsaI cleavage were designed in such a way that they are non-palindromic, non-compatible to each other and most importantly, do not produce compatible ends even if one or more bases of the overhangs are excised. Therefore, ligation of any insert carrying compatible ends would generate a recombinant without recreating the BsaI sites.

### Design and construction of the vector

The vectors (pVLExp) described in this work were derived from the T7 promoter-based *lac* operator (T7-Lac) expression vector pVNLEBAP1306 [13] carrying a pET11a backbone (Fig. 1). The pVNLEBAP1306 vector was modified by oligonucleotide-directed mutagenesis to delete the non-functional *tet^r^* gene between *laci* and the plasmid origin of replication and to also destroy a BsaI restriction site located in the β-lactamase gene. A 1.8 kbp stuffer flanked by two appropriately-oriented BsaI sites was cloned between the NdeI site (CATATG) that contains the initiation codon (underlined) and the EcoRI site. This stuffer was preceded by a sequence encoding Tag1 and a NheI restriction site upstream of the 5′ BsaI restriction site, and Bsu36I and Tag2 sequences downstream of the 3′ BsaI site, just before the stop codon. A series of expression vectors was constructed (Fig. 1 & Fig. S5) that contain different N- and/or C-terminal tags that can be used for purification purposes. The vector pVLExp4037 carries a deca-histidine (H10) as Tag1 so that after cloning an insert, the genes of interest are expressed with an N-terminal H10 tag and 5-amino acid spacer (GSGGG) to allow spatial accessibility of the tag for purification. The vectors pVLExp4038 and pVLExp4039 are similar and allow cloning of genes of interest to express proteins with an N-terminal poly-His and C-terminal Strep tag or with an N-terminal Strep tag and C-terminal poly-His, respectively. Prior to cloning, each vector was prepared by digestion with BsaI to remove the 1.8 kbp stuffer.

### Insert preparation and cloning strategy

Insert preparation involves PCR amplification of genes of interest using primers containing a 20–26 base-long gene-specific annealing sequence with an additional seven defined nucleotides at the 5′ end (Fig. 2C). To make the PCR product compatible with cloning, it was treated with T4 DNA polymerase in the presence of dTTP to create the four base-long overhangs 5′-CGGC and 5′-CTCC at the 5′ and 3′ ends of the insert, respectively (Fig. 2D). In this particular case, the 3′- exonuclease activity of T4 DNA polymerase would remove A, G and C bases and then stall when polymerase will encounter T because of the presence of dTTP in the reaction mix (due to equilibrium between the 3′-exonuclease and polymerase activities of the enzyme). These ends are compatible with the overhangs generated in the vector by digestion with BsaI (Fig. 2B). Thus, ligation of the linearized vector and T4 DNA polymerase-treated insert results in directional cloning of the insert to obtain a recombinant without recreating the BsaI sites (Fig. 2E).

However, before T4 DNA polymerase treatment, the insert has to be purified from the excess primers, dNTPs and polymerase used for amplification. Two rapid strategies were developed for this purpose. The first strategy (column method) involves use of silica-based columns for purification of the PCR product to remove excess primers, dNTPs and also the thermostable DNA polymerase. This method requires ∼2 µg amplified product (50–100 µl of PCR mix) for purification on the column. The subsequent step involves treatment of a fraction of the purified PCR product (250–500 ng in a volume of 10 µl) with T4 DNA polymerase in the presence of the appropriate buffer and dTTP in a total volume of 12.6 µl. The second strategy is a single tube method performed in a thermocycler. For this method, 10 µl of PCR mix (containing 250–500 ng crude amplified product) is first treated with a mixture of Exonuclease I (Exo I) and shrimp alkaline phosphatase (SAP), which is commercially available as Exo-SAP. Exo I degrades the primers and SAP dephosphorylates the released nucleotides and excess dNTPs present the PCR mix after amplification. Both of these enzymes are then heat-inactivated in the same tube, followed by addition of dTTP and T4 DNA polymerase without the need for adding any buffer, as T4 DNA polymerase is compatible with most buffers used for PCR. After 1 h incubation, the contents of the tube are heated to inactivate T4 DNA polymerase. Thus, using the single tube strategy, an insert compatible with cloning is prepared in the same tube without any need for transferring the amplified product.

Both insert preparation methods were initially evaluated by cloning four mycobacterial genes (*Rv1827, Rv3029c, Rv1077* and *Rv1908c*) of sizes, approximately 0.5, 1, 1.5 and 2.2 kbp. These inserts were amplified in 100 µl PCR reactions using either HPLC-purified or machine grade (unpurified) primers containing 24–26 base-long gene-specific annealing sequences along with a seven base-long additional sequence (Table S1). Then, 3 µl of each reaction mixture was analyzed by agarose gel electrophoresis; both types of primers yielded comparable amounts of amplified products, suggesting that the purity of the primers does not affect the quality and the quantity of the amplified insert (Fig. S1). The amplified products were processed to generate cloning-compatible inserts via both the single tube method and column method, as described in the *Materials and Methods*. In both cases, ligation was performed using 1 µl of insert (∼30 ng) and 25 ng of vector in a total reaction volume of 10 µl, and then 250 pg vector equivalent of the ligated sample was electroporated into competent *E. coli* cells.

Using column-purified inserts, ∼1– 5×10^6^ transformants per µg vector DNA were obtained. The number of transformants was not influenced by the size of the insert and nearly 100% of the clones obtained were recombinants (Table 1). Furthermore, DNA sequencing of the recombinant inserts demonstrated that over 90% of the clones contained the correct nucleotide sequences. Expression analysis of selected clones revealed that nearly all of the recombinants expressed proteins of the expected sizes (Fig. S2). The non-recombinant clones obtained were undigested vector sequences. The transformation efficiency of the ligation mixture prepared with inserts via the ‘single tube’ strategy was ∼5 times lower; however, over 95% of these transformants were recombinant. More than 80% contained the correct DNA sequences, and nearly 100% of clones expressed the desired proteins (Table 1 and data not shown). Thus, the ‘single tube’ strategy described in this paper is a simple, robust and rapid cloning method that requires small volumes of PCR amplified product and provides results comparable to the ‘column method’.

**Table 1:** Cloning efficiency of four genes using column method and single tube method.

Gene	Size in(bp)	Transformants/µgDNA^a^	Efficiency obtainedusing column method		Single tubemethod
			Recombinants/Totalamplified (%)	Number of proteinExpressingclones	Recombinants/Totalamplified (%)
Rv1827	496	∼3×10^6^	30/30 (100)	8/8	29/32 (91)
Rv3029c	1001	∼1×10^6^	31/32 (97)	3/4	32/32 (100)
Rv1077	1402	∼5×10^6^	28/29 (96.5)	8/8	29/30 (97)
Rv1908c	2230	∼1×10^6^	30/31 (97)	8/8	28/29 (96.5)

a The competent cells [BL21(DE3)RIL] used had efficiency of 1×10^8^ transformants/µg supercoiled pGEM3Z DNA.

**Figure 3:**
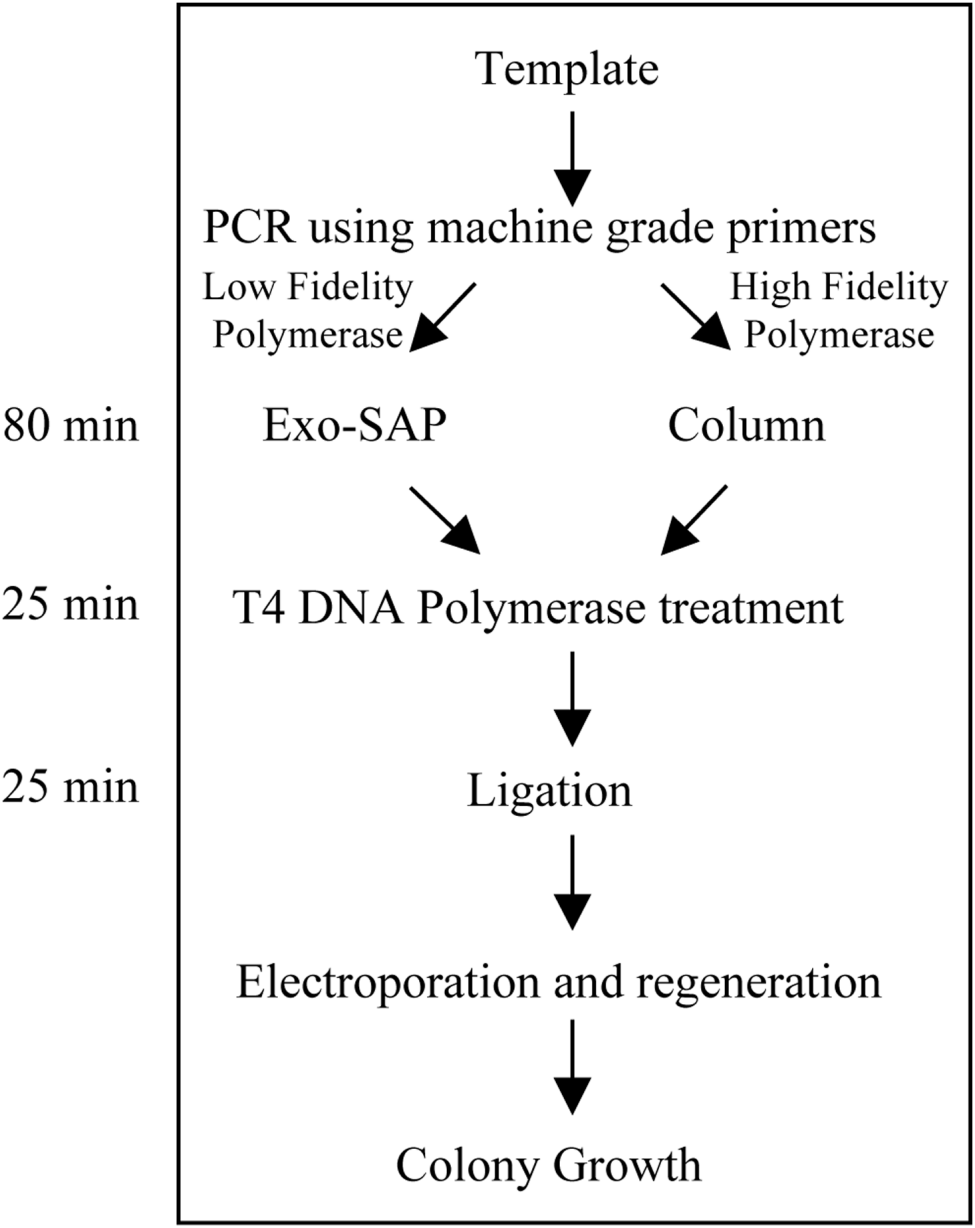
Flowchart and timing of the experimental steps for “Time Saver” cloning strategy.

To make the cloning strategy more rapid, such that the entire process from PCR to transformation could be completed in about 6–8 h, the timings of different treatment steps post-clean up (by either Exo-SAP treatment or column) were systematically evaluated using three PCR-amplified mycobacterial genes (*Rv1886c* [860 bp], *Rv1980c* [680 bp] and *Rv3763* [480 bp]; Fig. S3A). Eventually, the “time saver” cloning strategy was devised, in which the T4 DNA polymerase treatment time was reduced from 60 min to 5 min at 15°C and the ligation time was reduced from 2 h to 15 min at room temperature. Thus, after PCR clean up, the processing time up to transformation was reduced from 4 h to just 1.5 h. This reduction in treatment time did not affect the transformation efficiency, and 100% of the transformants obtained using the “time saver” cloning strategy were recombinants (Fig. S3B). The reproducibility of this new cloning strategy was further confirmed by cloning five different scFv genes into an arabinose promoter-based expression vector pVMAAscFvclo 0001. Analysis of the transformants obtained showed concordant results, as 100% of the clones screened were recombinants (Fig. S4). Thus, the “time saver” cloning strategy is a highly efficient and rapid method that can be completed within few hours post-PCR (Fig. 3).

More than 300 mycobacterial genes were cloned following the ‘single tube’ strategy using PCR products amplified with the Expand High Fidelity PCR System (Expand HF Polymerase) or Expand Long Template PCR System (Expand LT Polymerase). These enzymes are a cocktail of Taq DNA polymerase (low fidelity and high processivity) and Pwo DNA polymerase (high fidelity, lower processivity and high 3′ exonuclease activity). Since the amount of Pwo DNA polymerase in the cocktail is expected to be low (although unknown), the presence of 3′-exonuclease activity has no effect on the insert preparation process. However, the presence of Taq DNA polymerase creates PCR-borne mutations necessitating the use of other high fidelity and high processivity thermostable DNA polymerases to obtain error-free PCR products. However, one has to be careful while using high-fidelity enzymes with single tube cloning strategy described here. We presume that in the absence of dNTPs during Exo- SAP treatment, and at higher temperature required for its heat inactivation, the thermostable high-fidelity polymerases might exhibit uncontrolled 3′- exonuclease activity, which might remove much more bases than desired (i.e. only 4 bases). It should be noted that Exo-SAP-based clean-up of inserts in the ‘single tube method’ takes about 80 min and the ‘column method’ can produce PCR-amplified product free of polymerase, excess primers and dNTPs within the same time frame. The use of columns for clean-up does not necessarily take longer and up to 24 single PCR clean-up columns can be easily handled in a single batch within 80–90 min.

The proposed cloning strategy is highly versatile with the possibility of being used efficiently in either ‘single tube method’ suitable for the inserts amplified using thermostable polymerases with low/no 3′ exonuclease activity or ‘column method’ involving prior clean-up of the inserts amplified using a high fidelity thermostable polymerases with high 3′ exonuclease activity (Fig. 3).

### High-throughput application of the single tube strategy

The main objective of developing this novel cloning strategy was to clone and express the entire proteome of *Mtb*; therefore, the protocol was developed for high-throughput cloning and expression of genes. For high-throughput cloning, a batch of 24 genes was selected for ease of handling. As an example, 24 mycobacterial genes of various sizes ranging from 170 bp to 396 bp (Table 2) were amplified from *Mtb* genomic DNA using gene-specific primers and Expand HF Polymerase in the presence of 1% DMSO. The PCR products were processed using Exo-SAP clean-up and the single tube strategy to obtain inserts with four base-long overhangs. For each target gene, colony PCR and sequencing analysis indicated that more than 95% of the colonies were recombinants (Fig. S6). High-throughput expression analysis of selected clones revealed that most clones had a good level of expression (Table 2). Following the same strategy, 12 batches of 24 genes each (ranging from 200 bp to 1.5 kbp) were cloned with similar efficiencies (Table 3; data shown for selected clones).

**Table 2:** Cloning efficiency and expression data of 24 genes (174 to 396 bp) using single tube strategy.

S.No	Sample	Size in(bp)	Mol.wt.(kDa)	Recombinants/Totalamplified (%)^a^	ExpressionResults^b^
1.	Rv0666	174	9.8	8/8 (100)	N
2.	Rv3250c	183	10.7	6/6 (100)	Y
3.	Rv2803c	216	11.5	8/8 (100)	Y
4.	Rv1211	228	11.8	7/7 (100)	Y
5.	Rv1134	237	12.2	8/8 (100)	Y
6.	Rv1298	243	12.7	8/8 (100)	Y
7.	Rv1335	282	13.5	8/8 (100)	Y
8.	Rv1738	285	14.6	8/8 (100)	Y
9.	Rv0287	294	13.8	7/7 (100)	Y
10.	Rv2117	294	14.8	8/8 (100)	Y
11.	Rv3905c	312	14.4	8/8 (100)	Y
12.	Rv1579c	315	15.2	5/5 (100)	Y
13.	Rv3065	324	15.0	8/8 (100)	N
14.	Rv2348	327	15.3	8/8 (100)	N
15.	Rv2919c	339	16.2	7/7 (100)	Y
16.	Rv2007	345	16.0	7/7 (100)	Y
17.	Rv0253	357	16.4	5/5 (100)	Y
18.	Rv3748	360	16.6	7/8 (100)	N
19.	Rv2446c	372	17.3	8/8 (100)	N
20.	Rv3923c	378	17.9	7/8 (87.5)	Y
21.	Rv3289c	378	17.1	8/8 (100)	N
22.	Rv3675	378	17.7	7/7 (100)	Y
23.	Rv3346c	393	18.0	7/7 (100)	Y
24.	Rv1224	396	18.0	6/6 (100)	Y

a as determined by colony PCR.

b Y-Yes; N-No; all clones contained correct DNA sequence.

**Table 3:** Cloning efficiency and expression data of 12 genes (720–1320 bp) cloned using single tube strategy.

S.No	Sample	Size in(bp)	Mol.wt.(kDa)	Recombinants/Totalamplified (%)^a^	ExpressionResults^b^
1.	Rv2018	720	26.0	7/8 (87.5)	Y
2.	Rv2603c	756	26.8	6/6 (100)	Y
3.	Rv3814c	786	27.2	7/8 (87.5)	N
4.	Rv0877	789	27.4	7/7 (100)	Y
5.	Rv0765c	828	29.0	8/8 (100)	Y
6.	Rv2905	945	33.4	6/7 (85.7)	Y
7.	Rv1207	957	33.0	7/8 (87.5)	Y
8.	Rv2560	978	33.1	7/8 (87.5)	N
9.	Rv2837c	1011	35.4	8/7 (100)	Y
10.	Rv1415	1278	46.1	7/8 (87.5)	Y
11.	Rv3703c	1281	47.1	8/8 (100)	Y
12.	Rv2836c	1320	48.4	6/8 (75)	Y

a as determined by colony PCR.

b Y-Yes; N-No; all clones contained correct DNA sequence.

### Application for genome-scale library construction

The cloning strategy described in this paper is not only suitable for routine and high-throughput cloning, but was also employed to efficiently construct highly complex whole genome fragment libraries of *Mtb* [17]. For high efficiency cloning, the quality of the insert, particularly its purity and quantity, is highly critical for efficient ligation. Therefore, for the library scale protocol, ∼10 µg of amplified product was purified by the column method followed by treatment of ∼5 µg of the purified insert with T4 DNA polymerase in the presence of dTTP to create overhangs. The T4 DNA polymerase-treated insert was further column purified. Prior to ligation, the concentration and purity of the insert was estimated by gel electrophoresis, and then the insert was ligated to the BsaI-digested vector in 3-fold molar excess. The insert preparation has been described in Fig. S8 with necessary details about the procedure. Two large gene fragment libraries MTBLIB25C01 and MTBLIB27C01 containing 100–300 bp and 300–800 bp fragments, respectively, of the *Mtb* genome were constructed by cloning randomly generated fragments between the PelB signal sequence (*PelBss*) and full-length trypsin-resistant *gIIIP* in a phagemid based phage display vector (pVCEPI23764; Fig. S5B). The cloning efficiency ranged from 5×10^7^ to 1×10^8^ transformants per µg vector DNA using cells with a transformation efficiency of ∼5×10^9^ transformants/µg supercoiled DNA. The library of 100–300 bp fragments contained ∼5×10^7^ independent clones, while the library of 300–800 bp fragments contained ∼1×10^8^ independent clones. Sequence analysis of randomly-selected transformants from both libraries revealed that nearly 97% of the clones were recombinants with nucleotide sequences aligning to the *Mtb* genome [17].

## Discussion

The proposed cloning strategy for PCR-amplified DNA employs unique combination of the type IIs restriction endonuclease BsaI to create a linearized vector with four base-long 5′-overhangs, and T4 DNA polymerase treatment of the insert in presence of a single dNTP to create vector-compatible four base-long overhangs. Thus, the preparation of inserts with precise overhangs is restriction endonuclease-free.

The vector design, insert preparation and overall cloning strategy described here meets most of the essential criteria for an efficient and high-throughput cloning strategy to allow rapid cloning of PCR-amplified DNA and expression of the encoded proteins. The system includes the following advantages: (i) the reagents for vector and insert preparation are commonly used enzymes available from multiple sources, (ii) the four base-long overhangs are designed to be non-palindromic, preventing self-ligation of the empty vectors, (iii) crude primers without any modifications and carrying an only seven base-long extra sequence are used for amplification, avoiding the synthesis of long primers and introduction of numerous extra residues with large hydrophobic side chains into the final product, (iv) the insert preparation requires simple enzymatic treatment that allows directional cloning in the vector without concatemerization of the inserts, and (v) the vector can be easily customized. Furthermore, using the proposed cloning strategy, one can process an unlimited number of samples that can be cloned in a single day (Fig. S4) with a high cloning efficiency of nearly 100% error-free recombinants. The main challenge for the proposed strategy was its compatibility with PCR products amplified using high fidelity enzymes with high 3′-exonuclease activity. However, this issue was resolved by the use of column purification to remove the DNA polymerase, without significantly extending the processing time. We have successfully employed this single tube strategy and the column method to clone nearly 400 genes of various sizes (200 bp to 1.5 kbp) encoding proteins of diagnostic importance.

In recent years, several rapid cloning strategies based on the use of type IIs restriction enzymes have been described [11], [12]. However, these methods require digestion of the PCR product with restriction endonucleases, thus limiting their use to the inserts which are devoid of these sites [11]. Most methods require either purification of the amplified product or addition of chemicals to inhibit polymerase activity [12]. Additionally, the T4 DNA polymerase treatment has been used to generate compatible 3′ overhangs in the vector and insert in different ways; however, an overlapping sequence of >15 bp is needed, thus requiring long primers for amplification of the insert [4]. In LIC method, a specially designed vector is used where, in recombinants, the insert is flanked by extra nucleotides leading to extra amino acid residues. In SLIC strategy, although the vector compatible insert is prepared by PCR using long overhangs, the duration of treatment with T4 DNA polymerase might have to be carefully controlled [4]. This deficiency seems to have been resolved in SLiCE, but the requirement for long extra sequences to be added to the primers is a matter of concern [5]. In the proposed method for insert preparation, treatment with T4 DNA polymerase is independent of time, as 5–60 min treatment produces similar efficiencies. This has been possible due to the presence of one of the predefined dNTP in the insert preparation reaction. In the example shown in this paper, dTTP was used as single nucleotide because the overhangs in the vector backbone contained only A, G and C bases and therefore the compatible ends in the insert had T, G and C bases. Encounter of T base by exonuclease activity of the T4 DNA polymerase would terminate the exonuclease activity in the presence of dTTP. The presence of one or two of the four dNTPs to terminate the exonuclease would depend upon the overhangs in the vector and thus the sequence of overhangs in the insert. Thus, very clean and robust protocols described here to prepare vector and inserts each with precise four-base overhangs have resulted in the novel, simple and efficient cloning strategy, which is suitable for both high-throughput cloning of PCR-amplified DNA and also for high efficiency library scale applications.

The strategy of insert preparation by T4 DNA polymerase in the presence of single dNTPs can also be used to produce modules with compatible ends to ligate multiple fragments thus expanding the scope of previously described strategy which is based on the use of type IIs restriction endonucleases, which limits the use only to those modules that are devoid of those restriction sites [18], [19]. However, this process will require, either use of primers carrying 5′ phosphate group for amplification of DNA or phosphorylation of the amplified products before T4 DNA polymerase treatment.

Using our proposed strategy, it is theoretically possible to produce 256 different four base-long overhangs comprising three of the four bases, both in the vector and the insert. However, the sequences of the overhangs can be selected to avoid palindromic sequences that will not produce compatible ends, even if a few bases of the overhang in the vector become excised. In our proposed strategy, the sequences of the overhangs were selected to ensure this aspect in addition to incorporating sequences that encode for amino acids lacking side chains that provide minimum steric hindrance.

Thus, in summary, the proposed strategy for cloning PCR-amplified products is superior, less complex and highly versatile compared to previously described cloning strategies [11], [12], as existing strategies require either the use of restriction enzymes to prepare the insert or long primers for amplification and mandatory purification of the amplified inserts, and have not been demonstrated for constructing large libraries. The versatility of the newly described strategy is also evident from the following brief description of its possible applications and advantages. The basic vector pVLExp carrying the ColE1 origin of replication has been systematically modified to include additional features, as follows:

- The pVMExp series of vectors has been created by deleting the *rop* gene, which has increased the copy number to facilitate preparation of the plasmid. This change did not affect the basal expression level of proteins or the level of expression after induction. Fig. S7 shows the systematic changes that have been carried out to convert the pET11 backbone into smaller backbone of pVMExp vectors without loosing any function.
- The tags have been modified to contain a TEV protease site between the H10 tag and encoded protein. The H10 tag was also replaced with H10-MBP (maltose binding protein tag) and TEV protease site to improve the solubility of the expressed proteins. Protocols have been developed to remove the H10 and H10-MPB tags from the purified proteins to obtain tag-free proteins (pVL/pVMExp4337/pVMMBPExp4437).
- The pVMExp vector backbone has been further altered to replace the pBR322-derived ColE1 replicon with another origin of replication, such as CloDF13, RSF1030 or P15A, along with different antibiotic markers such as a chloramphenicol resistance gene or kanamycin resistance gene. The T7 promoter has been replaced by other promoters, such as the inducible *ara*BAD promoter (PBAD), tetracycline promoter (Ptet) or Lac promoter (Plac). Many of these new vectors have been used extensively for cloning *Mtb* toxin and anti-toxin genes and to carry out functional studies with the co-transformants by differential expression using arabinose and tetracycline [20]. The vectors mentioned above have also been used to clone and express several *Chandipura* virus proteins [21].
- The above-mentioned vectors are being modified to contain a stuffer encoding the *SacB* gene as a positive selection marker, with the aim of obtaining 100% recombinants.

Thus, the concept of creating 4-base overhangs using T4 DNA polymerase treatment in the presence of one or two specific dNTPs to create non-compatible, non-palindromic ends and enable efficient ligation to a BsaI-digested vector that carries compatible overhangs for directional cloning is already being practiced as a robust, simple, cost effective cloning strategy which has a high efficiency and is suitable for high-throughput applications.

## Supporting Information

Figure S1 **PCR amplification of four mycobacterial genes.** Amplification of genes (1) Rv1827 (2) Rv3029c (3) Rv1077 (4) Rv1908c using (a) HPLC grade primers and (b) Molecular Biology grade primers.
(TIF)

Figure S2 **Total cell expression of four mycobacterial genes by auto-induction method.** Total cell culture of different clones of each gene Rv1827 (lane 1–8), Rv3029c (lane 9–16), Rv1077 (lane 17–20) and Rv1908c (lane 21–28) were analyzed on 8–20% gradient gel by SDS-PAGE. The protein bands were visualized with Coomassie blue R-250 staining. Arrowhead indicates the band for different expressed proteins.
(TIF)

Figure S3 **PCR amplification of three mycobacterial genes.** (A) Amplification of three genes. (B) Colony PCR results of three genes amplified using T7P (5′ TAATACGACTCACTATAGGGGA 3′) and T7Tn (5′ CAGCCAACTCAGCTTCCTTTC 3′) primers. Eight clones were screened from each of the 3 mycobacterial genes cloned by colony PCR and analyzed on 1.2% agarose gel.
(TIF)

Figure S4 **Colony PCR results of five scFv genes amplified using AraP51 (5′ GCATTTTTATCCATAAGATTAGCG 3′) and T7Tn primers.** Eight clones were screened from each of the five scFv genes cloned by colony PCR and analyzed on 1.2% agarose gel.
(TIF)

Figure S5 **The figure above summarizes features of the various vectors constructed for employing restriction enzyme-free cloning strategy.** Figure A1–A5, pVMExp T7-promoter based vectors for protein expression; B, pVC vector for constructing gene fragment libraries for display on filamentous phages; C, pRAK/pCAK Arabinose promoter (*ara*BAD) based expression vectors.
(TIF)

Figure S6 **Colony PCR results of 24 genes amplified using T7P and T7TN primers.** Eight clones were screened from each of the 24 mycobacterial genes cloned (1/24 to 24/24) by colony PCR and then analyzed on 1.2% agarose gel.
(TIF)

Figure S7 **Schematic representation of the modifications carried out in pET11a for construction of pVMExp vectors.** The numbers in the brackets represent the size of the vector backbone (in bp) excluding the sequence located between T7 promoter-lac operator (T7lac) and T7 terminator (T7TN).
(TIF)

Figure S8 **Insert preparation for library- scale cloning.** Genomic DNA of *M. tuberculosis* H37Rv was fragmented by sonication to obtain fragments in the range of 100–1200 bp (A). The fragments of sizes differing by increments of 100 bp (eg. 100–200 bp, 200–300 bp) were purified from 1.2% Sea Plaque GTG agarose (Lonza, Rockland, ME, USA). Two mixtures of fragments were prepared namely, ‘100–300 bp’ mix, which was prepared by mixing 100–200 and 200–300 bp fragments in molar ratio- 1∶1.5, and ‘300–800 bp’ mix, which was prepared by mixing 300–400, 400–500, 500–600, and 700–800 bp fragments in molar ratio- 1∶1.5∶2∶2.5. The larger fragments were added in higher molar ratios to compensate for their ligation efficiencies. 10 µg of each fragment mix was end-repaired and phosphorylated (B) using Quick Blunting kit (NEB) followed by ligation of two adaptors (C) with blunt end on one side but 4-base 5′ overhang on the other side to achieve directionality during adapter ligation. 5′ (D1) and 3′ (E1) adapter duplex were 34 and 33 bp long, respectively, encoding for sequences that served as spacer. The sense strand of D1 carried 5′ biotin. Adapter ligation was set up using 10 µg mixture of fragment with 30 mole excess each of adapter duplex D1 and E1. Following ligation, the unligated adapters were removed by Qiaquick PCR purification kit (Qiagen) followed agarose gel electrophoresis. The adapter ligated DNA was treated with BstI DNA polymerase (D), followed by isolation of single stranded DNA (ssDNA) carrying one strand of D1 and E1 adapters on either end of a fragment using streptavidin coated M280 beads magnetic beads (E). These steps were similar to the process described for pyrosequencing template preparation [22]. In the process here, the ssDNA was then subjected to PCR amplification using primers, which anneal to D1 and E1 sequences (F), and amplified double stranded DNA (dsDNA) was obtained using 1∶40 dilution of single stranded DNA for 20 cycles in 50 ul reaction. After clean up ∼5 µg dsDNA was treated with T4 DNA polymerase in the presence of dTTP, which resulted in 4- base long 5′ overhangs, CGGC and CCTC at the 5′ and 3′ ends of the fragments (H). the part sequence (7 bases) of the adapter is shown in H and I as the remaining portion of the adapter can vary depending upon the requirement. These two ends are compatible with BsaI-digested vector pVCEPI23764. The library was constructed by ligating 2 µg BsaI-digested vector with 3 molar excess of two types of fragment mixtures in total volume of 100 µl, which were incubated for 16 hrs at 16°C (J). The entire ligation reaction was electroporated in *E.coli* TOP10F’ cells (4 µl ligation mix/100 µl cells). This produced CO1 cells (K) as described earlier [17]. The library sizes have been described in the methods.
(TIF)

Table S1 **Primers used for the amplification of mycobacterial genes.**
(TIF)
