# Supplementary figures and images for: Rapid Restriction Enzyme-Free Cloning of PCR Products: A High-Throughput Method Applicable for Library Construction
Source: PLoS One. 2014 Oct 31;9(10):e111538. doi: 10.1371/journal.pone.0111538 (PMC4216109; doi:10.1371/journal.pone.0111538)

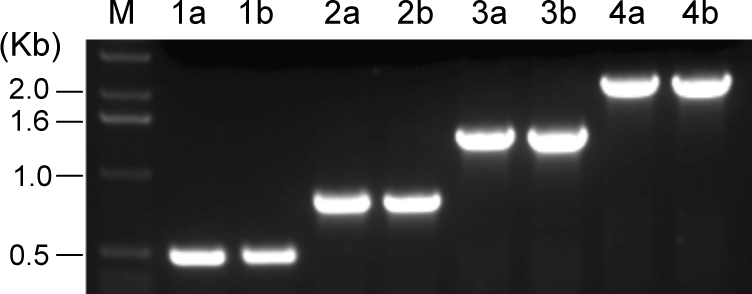

Supplement: Figure S1 — PCR amplification of four mycobacterial genes. Amplification of genes (1) Rv1827 (2) Rv3029c (3) Rv1077 (4) Rv1908c using (a) HPLC grade primers and (b) Molecular Biology grade primers. (TIF) [file pone.0111538.s001.tif]

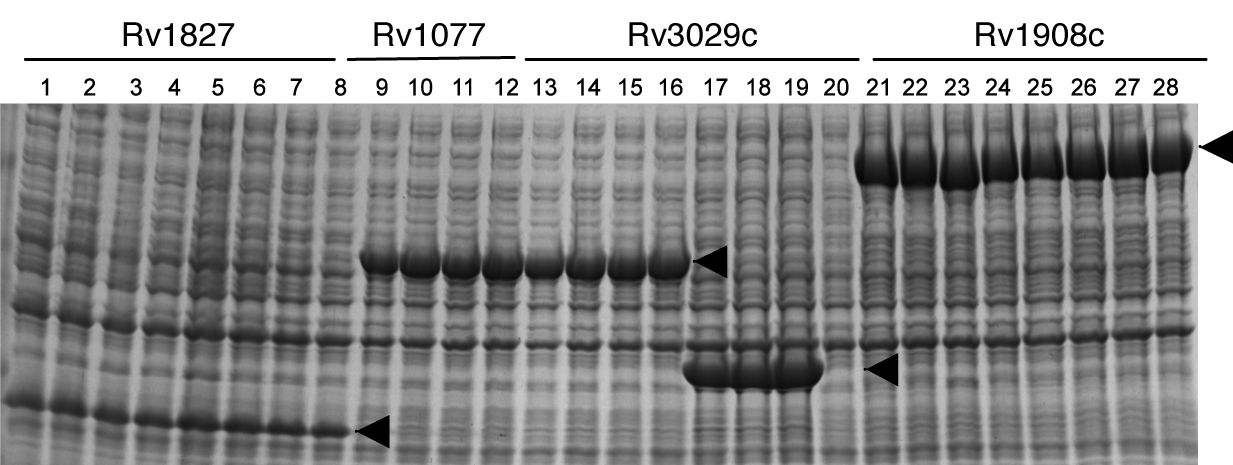

Supplement: Figure S2 — Total cell expression of four mycobacterial genes by auto-induction method. Total cell culture of different clones of each gene Rv1827 (lane 1–8), Rv3029c (lane 9–16), Rv1077 (lane 17–20) and Rv1908c (lane 21–28) were analyzed on 8–20% gradient gel by SDS-PAGE. The protein bands were visualized with Coomassie blue R-250 staining. Arrowhead indicates the band for different expressed proteins. (TIF) [file pone.0111538.s002.tif]

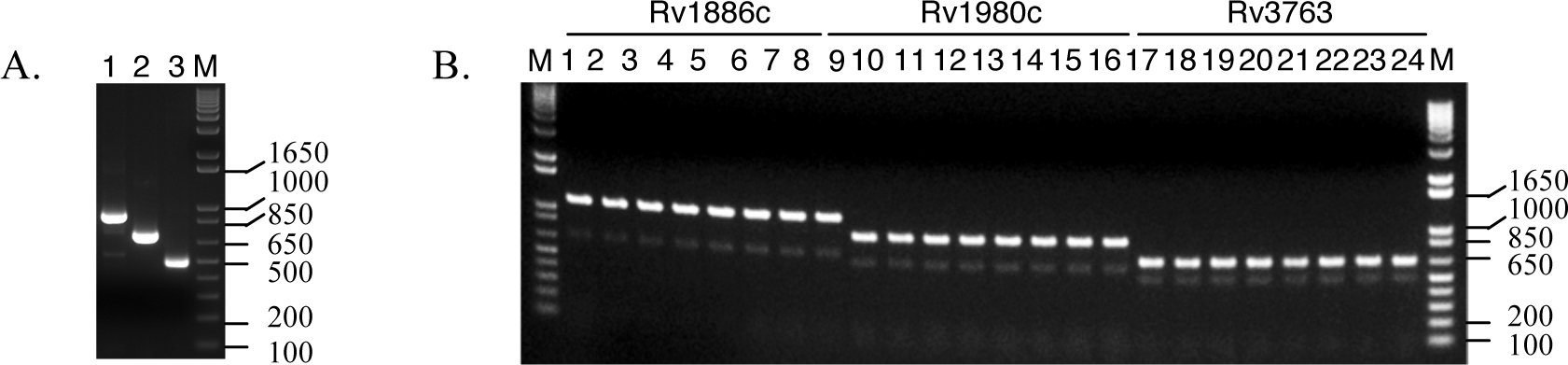

Supplement: Figure S3 — PCR amplification of three mycobacterial genes. (A) Amplification of three genes. (B) Colony PCR results of three genes amplified using T7P (5′ TAATACGACTCACTATAGGGGA 3′) and T7Tn (5′ CAGCCAACTCAGCTTCCTTTC 3′) primers. Eight clones were screened from each of the 3 mycobacterial genes cloned by colony PCR and analyzed on 1.2% agarose gel. (TIF) [file pone.0111538.s003.tif]

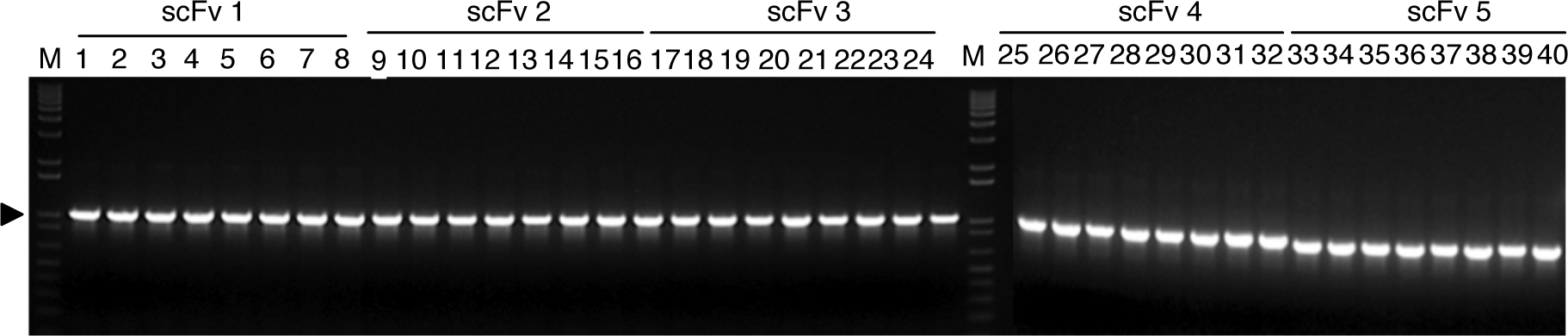

Supplement: Figure S4 — Colony PCR results of five scFv genes amplified using AraP51 (5′ GCATTTTTATCCATAAGATTAGCG 3′) and T7Tn primers. Eight clones were screened from each of the five scFv genes cloned by colony PCR and analyzed on 1.2% agarose gel. (TIF) [file pone.0111538.s004.tif]

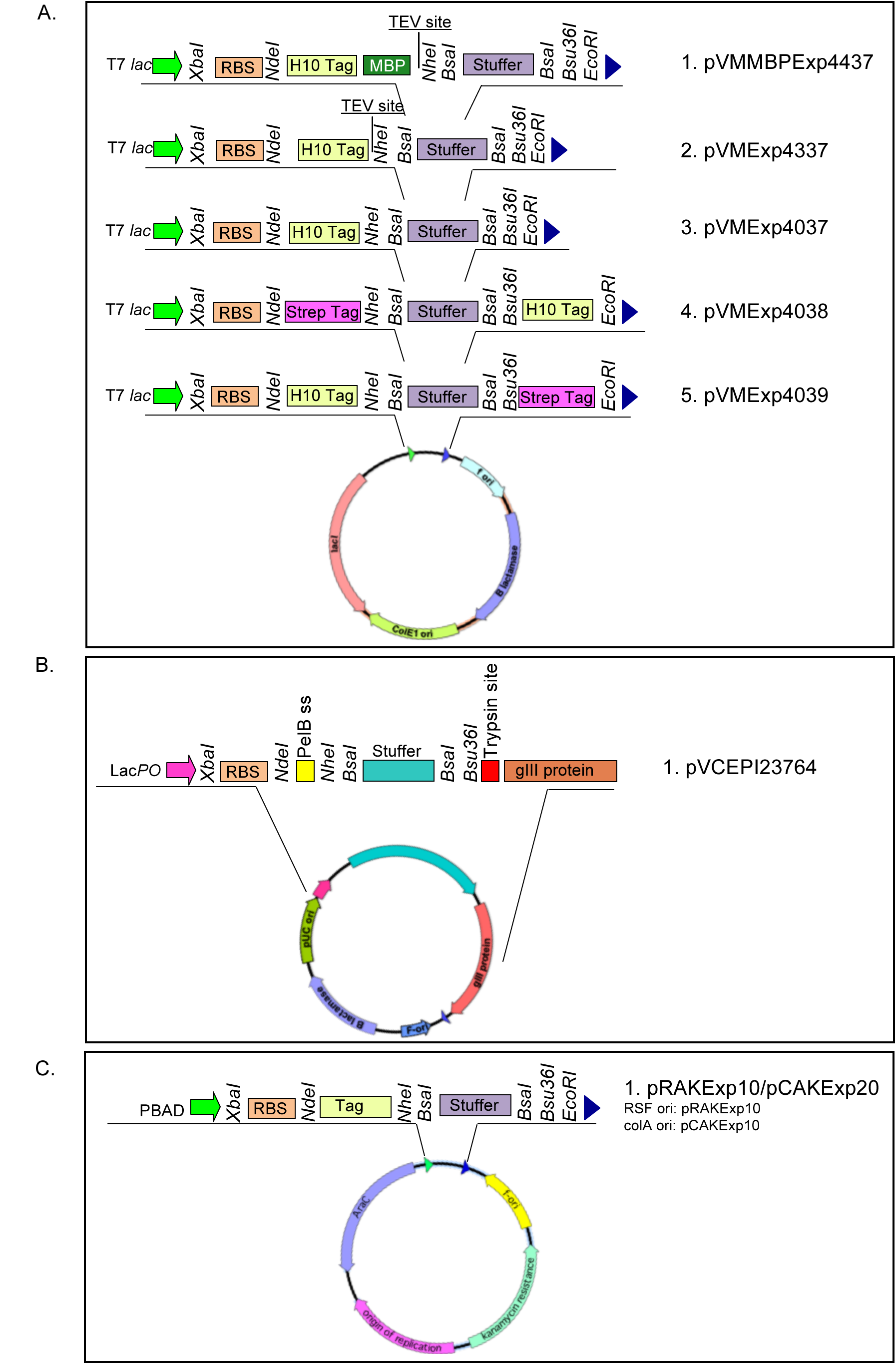

Supplement: Figure S5 — The figure above summarizes features of the various vectors constructed for employing restriction enzyme-free cloning strategy. Figure A1–A5, pVMExp T7-promoter based vectors for protein expression; B, pVC vector for constructing gene fragment libraries for display on filamentous phages; C, pRAK/pCAK Arabinose promoter (araBAD) based expression vectors. (TIF) [file pone.0111538.s005.tif]

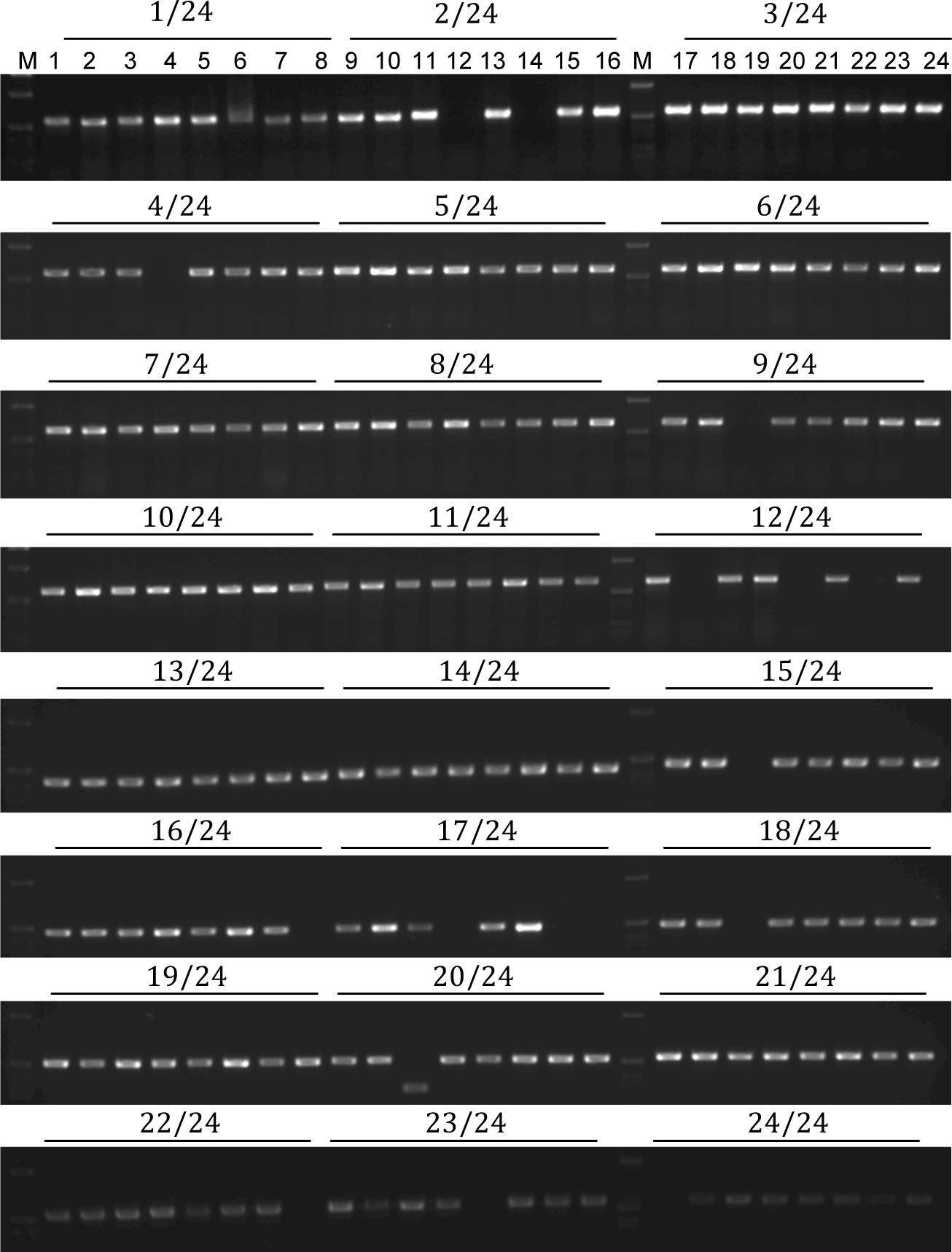

Supplement: Figure S6 — Colony PCR results of 24 genes amplified using T7P and T7TN primers. Eight clones were screened from each of the 24 mycobacterial genes cloned (1/24 to 24/24) by colony PCR and then analyzed on 1.2% agarose gel. (TIF) [file pone.0111538.s006.tif]

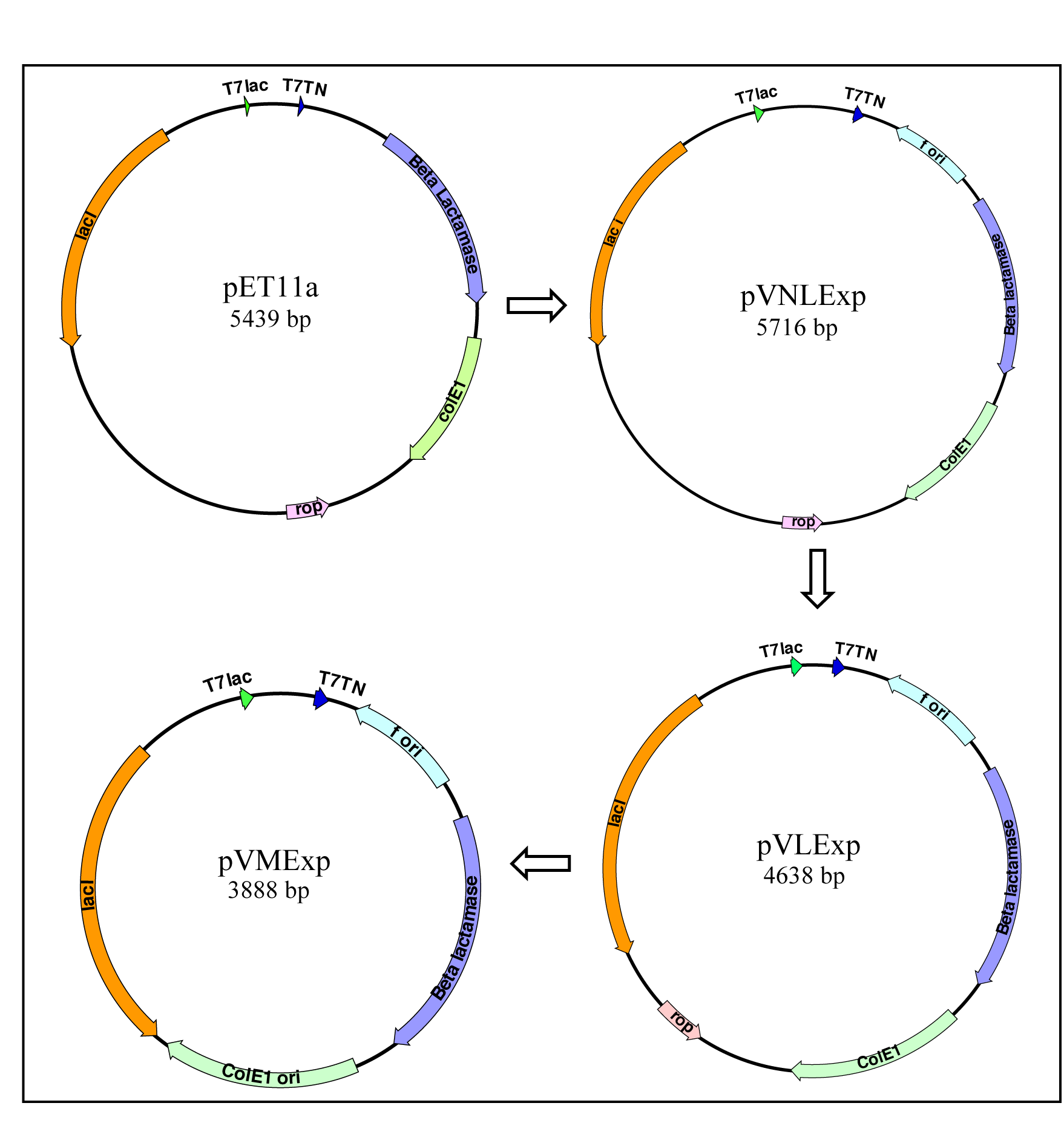

Supplement: Figure S7 — Schematic representation of the modifications carried out in pET11a for construction of pVMExp vectors. The numbers in the brackets represent the size of the vector backbone (in bp) excluding the sequence located between T7 promoter-lac operator (T7lac) and T7 terminator (T7TN). (TIF) [file pone.0111538.s007.tif]

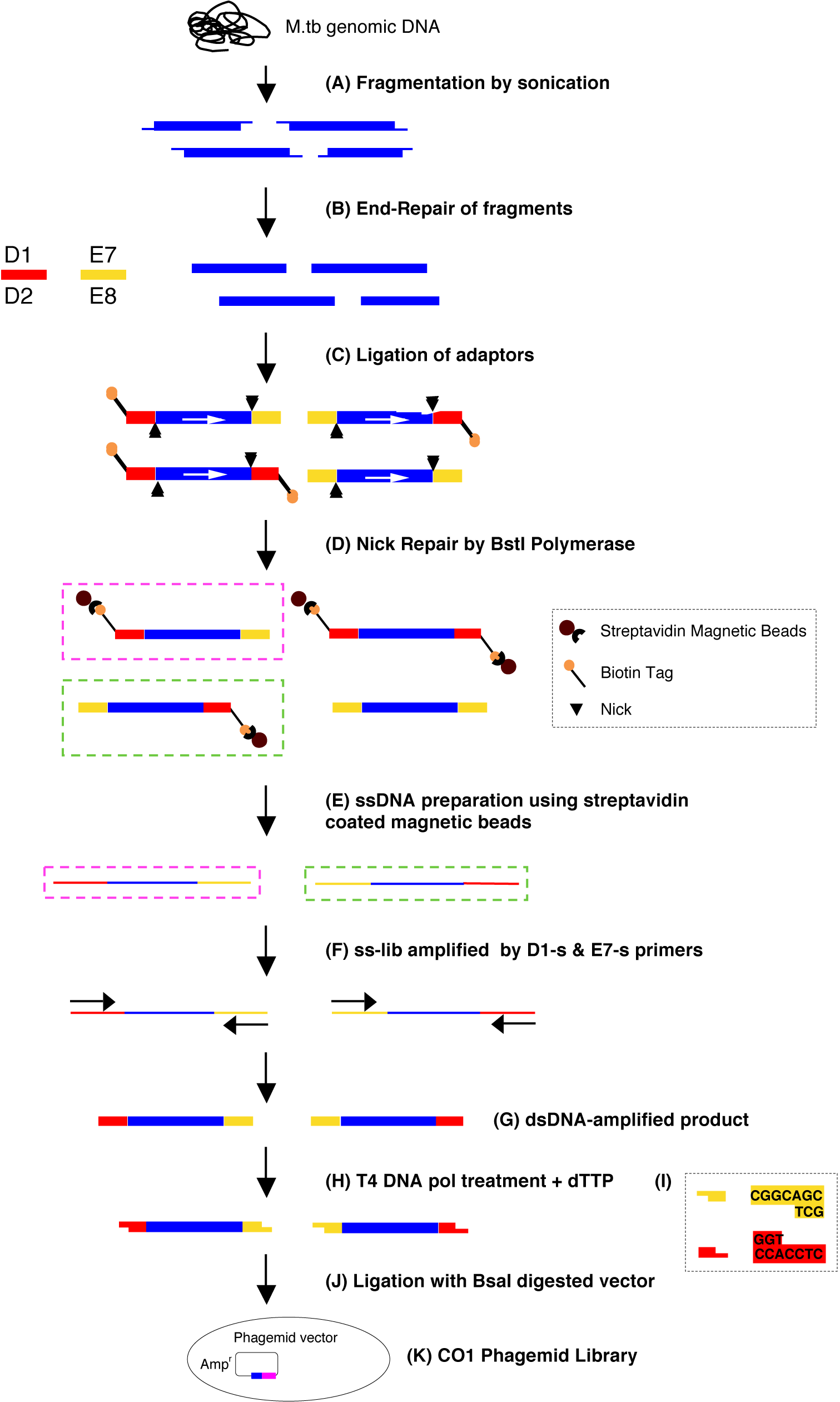

Supplement: Figure S8 — Insert preparation for library- scale cloning. Genomic DNA of M. tuberculosis H37Rv was fragmented by sonication to obtain fragments in the range of 100–1200 bp (A). The fragments of sizes differing by increments of 100 bp (eg. 100–200 bp, 200–300 bp) were purified from 1.2% Sea Plaque GTG agarose (Lonza, Rockland, ME, USA). Two mixtures of fragments were prepared namely, ‘100–300 bp’ mix, which was prepared by mixing 100–200 and 200–300 bp fragments in molar ratio- 1∶1.5, and ‘300–800 bp’ mix, which was prepared by mixing 300–400, 400–500, 500–600, and 700–800 bp fragments in molar ratio- 1∶1.5∶2∶2.5. The larger fragments were added in higher molar ratios to compensate for their ligation efficiencies. 10 µg of each fragment mix was end-repaired and phosphorylated (B) using Quick Blunting kit (NEB) followed by ligation of two adaptors (C) with blunt end on one side but 4-base 5′ overhang on the other side to achieve directionality during adapter ligation. 5′ (D1) and 3′ (E1) adapter duplex were 34 and 33 bp long, respectively, encoding for sequences that served as spacer. The sense strand of D1 carried 5′ biotin. Adapter ligation was set up using 10 µg mixture of fragment with 30 mole excess each of adapter duplex D1 and E1. Following ligation, the unligated adapters were removed by Qiaquick PCR purification kit (Qiagen) followed agarose gel electrophoresis. The adapter ligated DNA was treated with BstI DNA polymerase (D), followed by isolation of single stranded DNA (ssDNA) carrying one strand of D1 and E1 adapters on either end of a fragment using streptavidin coated M280 beads magnetic beads (E). These steps were similar to the process described for pyrosequencing template preparation [22]. In the process here, the ssDNA was then subjected to PCR amplification using primers, which anneal to D1 and E1 sequences (F), and amplified double stranded DNA (dsDNA) was obtained using 1∶40 dilution of single stranded DNA for 20 cycles in 50 ul reaction. After clean up ∼5 µ [file pone.0111538.s008.tif]

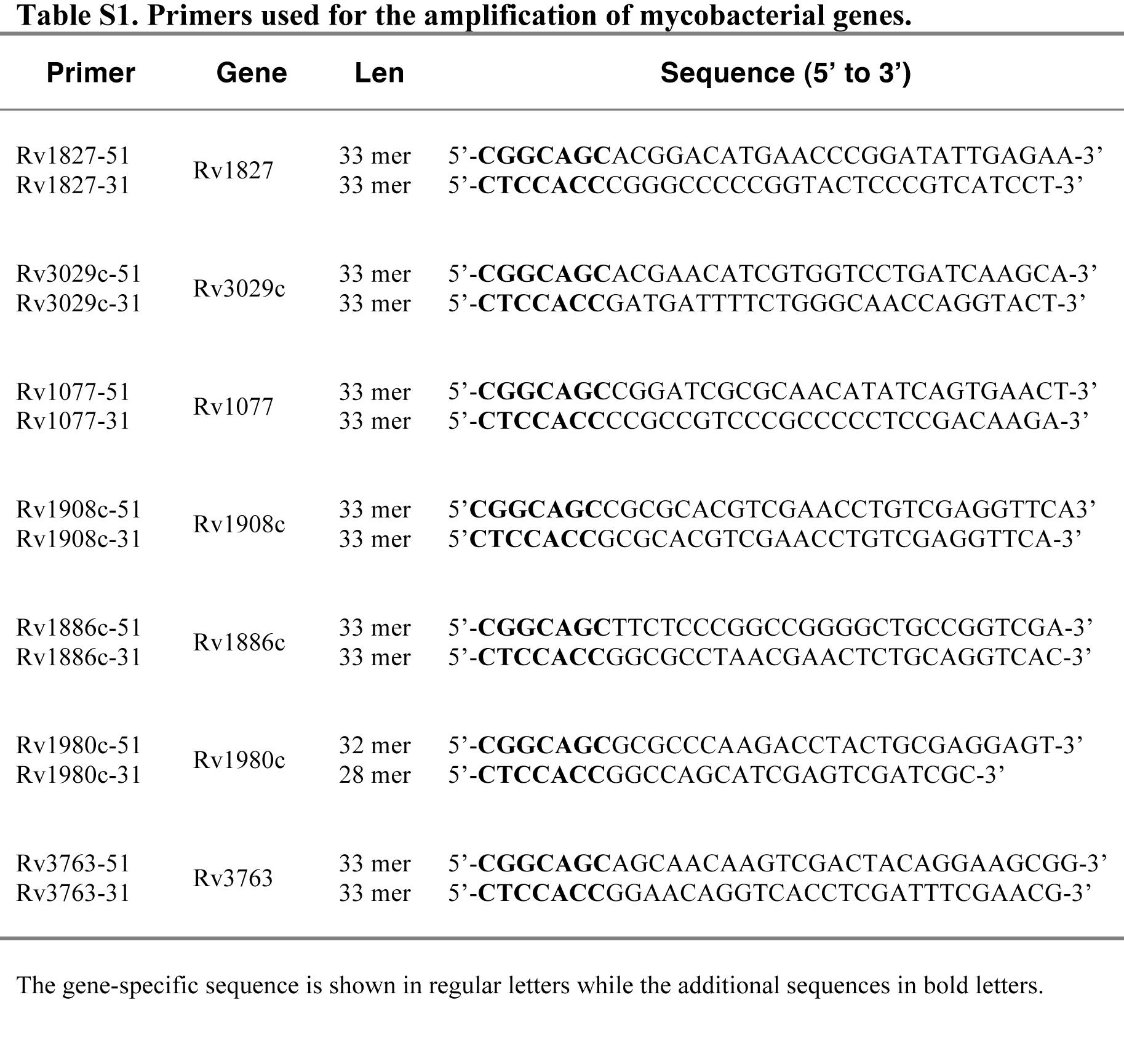

Supplement: Table S1 — Primers used for the amplification of mycobacterial genes. (TIF) [file pone.0111538.s009.tif]
